# Predictors of loss to follow up among adults on antiretroviral therapy before and after the start of treat-all strategy in public health facilities of Hawassa city, Ethiopia: A Competing risk regression

**DOI:** 10.1371/journal.pone.0299505

**Published:** 2024-03-14

**Authors:** Abera Gezume Ganta, Ermias Wabeto, Worku Mimani Minuta, Chala Wegi, Tezera Berheto, Serawit Samuel, Desalegn Dawit Assele

**Affiliations:** 1 Department of Public Health, College of Health Science and Medicine, Jinka University, Jinka, Ethiopia; 2 School of Public Health, College of Health Sciences and Medicine, Wolaita Sodo University, Wolaita Sodo, Ethiopia; 3 Department of Public Health, College of Medicine and Health Sciences, Hawassa University, Hawassa, Ethiopia; Jimma University, ETHIOPIA

## Abstract

**Background:**

Treat-all strategies improved patient outcomes, despite higher rates of loss to follow-up compared to the pre-treat era. Patients in Ethiopia experienced a higher rate of LTFU during the treat-all strategy period; however, studies did not identify contributing factors in comparison with previous strategies. This study aimed to assess the incidence and predictors of loss to follow-up before and after the start of the treat-all strategy among adults on anti-retroviral therapy in public health facilities in Hawassa City, Ethiopia.

**Methods:**

An institution-based retrospective follow-up study was conducted among 1190 randomly selected adults on antiretroviral therapy in public health facilities in Hawassa City. Using the Open Data Kit (ODK), data were collected from medical records and exported to Stata version 16 and R 4.2.1 for analysis. A Grays test and cumulative incidence curve were used to compare the cumulative incidence function of loss to follow-up. Bivariable and multivariable competing risk regression were fitted to identify predictors of LTFU and variables with a p-value <0.05 were considered significant.

**Results:**

The cumulative incidence of lost-to-follow-up was 4.92(3.84,6.3) and 8.67(7.26,10.3) per 100 person-years (PY) in pre-treat all and treat all cohorts, respectively. The cumulative incidence of mortality was 5.86(4.67,7.35) and 3(2.26,4.12) per 100 PY in pre-treat and treat all cohorts, respectively. Fair/poor adherence (aSHR:5.17; (95% CI 1.97, 13.51), underweight (aSHR:2.13; 95% CI: 1.15–3.93) and WHO stage III/IV (aSHR:2.69; 95% CI: 1.27, 5.71) were predictors of loss up in pre—treat all, whereas fair/poor adherence (aSHR = 2.07; 95% CI: 1.18, 3.68), underweight (aSHR:1.71; 95% CI: 1.13, 2.56), and CD4 cell >350 cell/m3 (aSHR: 1.67; 95% CI: 1.05, 2.65) predicts of loss up in treat all cohorts.

**Conclusion:**

This study demonstrated that the incidence of loss to follow-up was considerably higher in the treat-all period as compared to the pre-treat-all era. Poor medication compliance, underweight, and a CD4 level >350 cells/m3 contributed to the higher rate of LTFU in the treat-all strategy. Targeted interventions, such as nutritional support and strengthening medication adherence counseling, should be implemented to maintain treatment retention and reduce antiretroviral therapy dropout rates.

## Introduction

Loss to follow-up(LTFU) is defined as the failure of HIV-positive patients on medication refill follow-up visits for three or more months from the last appointment date and has not been documented as dead or transferred out to another HIV clinic [1]. The advantages of anti-retroviral therapies are compromised by LTFU, which also raises the risk of morbidity, death, and hospitalization due to AIDS [2–4]. It serves as a warning indicator of drug resistance, ineffective therapy, and mortality driven by suboptimal ART adherence [5, 6]. Antiretroviral therapy (ART) significantly decreases the viral load when people with HIV (PLHIV) adhere to their treatment schedule in full and for the rest of their lives [7].

Though LTFU has become a major public health concern for the ART program in developing countries [8, 9]. In sub-Saharan Africa and Asia-Pacific, more than 20% of patients fail to return to ART for more than six months annually [10]. The incidence of LTFU varies by region and setting in Ethiopia [3, 4, 11]. It was 3.7 per 100 PYO at Debre Markos [11] and 23.76 per 100 PY at Karmara General Hospital Jijiga town, Ethiopia [12]. The LTFU rate in the first six months was 15%, which significantly increased to 21% at the 12-month follow-up [13, 14].

Studies reported that several factors, such as sociodemographic factors, age [4, 11, 15], sex [16, 17], occupation [18], residence [19, 20], marital status [20], educational status [21], disclosure status [22], caregiver [17, 23, 24] and clinical and treatment-related factors like baseline WHO stage [3, 13, 25], body mass index [4], baseline CD4 count [26–28], opportunistic infection at enrollment [29], baseline functional status [30], not receiving prophylaxis [12, 29] and ART regimen [16] were predictors of LTFU.

Ethiopia implemented a universal strategy in January 2017 to enroll all HIV-infected patients in ART services [31]. Although there is sufficient data to support the benefits of starting ART early, which include increased uptake and care coordination, reduced HIV-related morbidity, slowed disease progression, and decreased HIV transmission [32], there were conflicting results regarding the association with obtaining satisfactory ART retention and optimal therapy adherence [22, 33]. To achieve the UNAIDS goals of 95-95-95, particularly the 95% viral suppression in ART-receiving populations, retention in care is essential. To end the HIV epidemic by 2030, further studies and interventions on LTFU and its contributing factors are crucial [34].

Ethiopia’s health ministry is implementing strategies to reduce follow-up rates, including standardized monitoring systems, reliable outcomes, early mortality reduction, uninterrupted drug supplies, non-toxic ART regimens, decentralization of care, reduced indirect costs, and strengthening links between health services and communities [35]. Despite government efforts, the LTFU rate remains high in the treat-all era [12], with no study conducted on it compared to the pre-treat-all era, necessitating ongoing efforts to provide updated information for effective test and treat strategies. Besides, in Ethiopia, studies on LTFU use standard Cox regression analysis [15, 36], which overlooks competing events, leading to an overestimation of LTFU incidence [37]. Fine and Gray competing-risks regression can reduce biases in estimating LTFU in competing event situations, as it considers death as a competing event [38, 39]. Thus, this study aimed to estimate the incidence and identify predictors of LTFU while considering death as a competing event in the public health facilities of Hawassa City, southern Ethiopia.

## Methods

### Study area and period

The study was conducted at public health facilities in Hawassa City from May 1 to 30, 2022. Hawassa City is located 273 Km far south of Addis Ababa, the capital city of Ethiopia. Currently, three governmental hospitals (Hawassa University Comprehensive Specialized Hospital, Adare General Hospital, and Tula Primary Hospital) and three health centers (Bushulo, Millennium, and Tula Health Center) are accessible for antiretroviral therapy services in Hawassa City. According to the City Health Department Health Information Management System (HIMS) report, there were 6095 HIV-infected people on ART follow-up in the health facilities at Hawassa City.

### Study design and population

An institution-based retrospective follow-up study was conducted at Hawassa City public health facilities from January 1, 2012, to December 31, 2021. All HIV-infected adults who enrolled at six public health facilities ART clinics in Hawassa City during the study period were the source population. All randomly selected HIV-infected adults who were enrolled at six public health facilities and ART clinics in Hawassa City were included in the study. Whereas, HIV patients with incomplete records regarding baseline characteristics (age, undefined outcome, date of ART initiation, and LTFU) and those who enrolled in ART through the prevention of mother-to-child transmission (PMCT) program were excluded.

### Sample size calculation and sampling procedure

The sample size was calculated based on the double population formula using Epi- info 7.2.1.0 considering the following assumptions: 95%CI, power 80%, the ratio of unexposed to exposed1:1, loss to follow up among rural residence = 13.7% and hazard ratio = 0.6 [11]. The final sample size after adding a 10% for chart incompleteness rate was 1190. By using proportional allocation, 434, 556,26,49,29 and 96 patients’ records were selected from Adare General Hospital, Hawassa University Comprehensive Specialized Hospital, Tula Hospital, Millennium Health Center, Tula, and Bushulo Health Center, respectively. STATA version 16 was used to generate a random sample. Subsequently, 1190 patient cards were selected at random using the subsequent unique number from the database registry.

### Variables of the study

The outcome variable in this study was the incidence of LTFU with death as a competing event. The predictor variables assessed were baseline socio-demographic factors (sex, age, marital status, educational status, occupation, residence, functional status, and disclosure status) and baseline clinical and treatment-related factors (baseline opportunistic infection, baseline CD4 count, type of regimen at the start, isoniazid and co-trimoxazole preventive therapy, baseline and last known WHO clinical stage, BMI and comorbidity).

### Operational definitions of variables

**Event**: loss to follow-up (LTFU) defined as not taking an ART refill for three months or longer from the last attendance for the refill and not yet classified as dead or transferred out [1]. **Censored**: individuals who were formally transferred- out to other health institutions after initiating ART and or individuals who remain active on ART follow-up at the end of the study.

**Drug adherence**: means taking the drug according to instructions given by the providers and estimated using three levels [40]. **Good—**equal to or greater than 95% adherence i.e., missing 1 dose from 30 doses or 2 out of 60 doses. **Fair**—85–94% adherence, i.e., missing 2–4 tablets from 30 tabs or 4 to 9 out of 60 doses. **Poor**—less than 85% adherence, i.e., missing more than five tablets from 30 tabs or > 10 tabs from 60 tablets.

### Data collection methods and tool

The data extraction tool was developed from the WHO standardized ART entry and follow-up chart, which is currently in use in ART clinics [4, 36, 41]. The entire cohort was organized into two separate cohorts of PLHIV based on the ART initiation date. These are pre-treating all cohorts (Jan1/2012 to Dec 31/2016) and treating all cohorts (Jan1/2017 to Dec 31/2021) which corresponds to the period before and after the start of implementation of the treat-all strategy. The ART register, SMART care, and patient follow-up card were used to determine the clients’ status. It was recorded as being dead (from whatever cause), lost and dropped, stopped, or retained in care. The data were collected from the patient charts by six BSc nurses and two supervisors using ODK.

### Data quality control

To control the data quality, the data extraction tool was pretested on 5% of the sample. The data collectors and the supervisor were trained on how to extract the data based on the study objectives. The data were checked for consistency and completeness daily by the supervisor and principal investigator. The codebook contained data cleaning for any missing values or data errors.

### Data processing and analysis

Data were collected by using ODK version 3.4 and then exported Stata 16 and R 4.2.1 software for analysis. Descriptive summaries like median, proportion, and graphs were used to describe baseline sociodemographic characteristics and clinical characteristics. A competing regression analysis was fitted to identify predictors of LTFU. Variables in the bi-variable proportional sub-distribution hazard model with a p-value ≤ 0.25 were included in the final multivariate analysis. The proportional sub-hazards assumption was checked using the global test, and it was met because it was 0.4304 and 0.845 in the pre-treat and treat all cohorts, respectively. Gray’s test and cumulative incidence curve were employed to compare the cumulative incidence function of LTFU. Variables with p-value < 0.05 were considered significant predictors of LTFU.

### Ethical consideration

Ethical clearance was obtained from the ethical review committee of the College of Health Sciences of Wolaita Sodo University (Ref No: CRCSD86/02/2014). A formal letter including the study objectives was submitted to the health departments of Hawassa City and HUCSH and permission was obtained from the City health department, the respective hospitals, and the health centers where the study was conducted. As the study was conducted through a review of patient records, no consent was obtained from the patients. Information about specific personal identifiers like the patient names was not collected and the personal informant was kept confidential throughout the study process.

## Results

### Baseline socio-demographic characteristics

A total of 1145 (96.2%) patient charts were included in the analysis; the remaining 45 (3.78%) were excluded due to eligibility criteria. The entire cohort was divided into 565 (49.3%) pre-treat-all cohorts and 580 (50.7%) treat-all cohorts. Nearly sixty percent (678) of the study participants were females. A large proportion of participants 196(34.89%) of pre-treat all cohort and 183(31.55%) of treat all cohort patients were in the age category of 25–34 years. The median age of 35 years (IQR = 28 to 40) and 32 years (IQR = 25 to 42.5) in pre-treat and treat all cohorts, respectively. The majority of the study participants 359 (63.54) of pre-treat all and 561(96.72) of treat-all cohorts were from urban and there were not many differences in socio-demographic backgrounds of clients between the two cohorts (Table 1).

**Table 1:** Baseline socio-demographic backgrounds of adult HIV patients on ART before and after the start of treat-all strategies in Hawassa City between Jan 2012, and Dec 2021.

Variables	Category	Pre-treat all n = 565	Treat all n = 580	p-value
Sex	Male	201(35.58)	266(45.86)	**< 0.001**
	Female	364(64.42)	314(54.14)	
Residence	Urban	359 (63.54)	561(96.72)	**< 0.001**
	Rural	206(36.46)	19(3.28)	
Type of facility	Hospital	472 (83.54)	499 (86.03)	0.954
	Health center	93(16.46)	81(13.97)	
Age	15–24	86(15.22)	137(23.62)	**< 0.001**
	25–34	196(34.89)	183(31.55)	
	35–44	194(34.69)	146(25.17)	
	>/ = 45	89 (15.75)	114(19.66)	
Marital status	Single	136(24.07)	210(36.21)	**< 0.001**
	Married	331(58.58)	290 (50)	
	Divorced	71(12.57)	68 (11.72)	
	Widowed	27(4.78)	12 (2.07)	
Educational status	No education	75(13.27)	59(10.17)	0.420
	Elementary	137(24.25)	242(41.72)	
	Secondary	146(25.84)	98(16.9)	
	College& above	207(36.64)	181(31.21)	
Occupation	Daily labourer	138(24.42)	148(25.52)	0.359
	Merchant	59(10.44)	68(11.72)	
	Student	143(25.31)	123(21.21)	
	Employer	105(18.58)	136(23.45)	
	Housewife	83(14.69)	72(12.41)	
	Others	37(6.55)	33(5.69)	
Disclosure	Yes	452(80)	530(91.38)	**<0.001**
	No	113(20)	50(8.62)	
Caregiver	Yes	446(78.94)	454(78.28)	0.785
	No	119 (21.06)	126 (21.72)	
Cell phone	Yes	444(78.58)	519(89.48)	**0.048**
	No	121(21.42)	61 (10.52)	

### Baseline clinical and treatment-related characteristics

The proportion of PLHIV commencing ART with CD4> 350 cells/increased from pre-treat all period to treat all period (5.84% vs. 53.97%, p < 0.001). The median baseline CD4 count of 195 (IQR = 126–310) in pre-treat all cohorts was lower than 452 (IQR = 342–550) in treat all cohort participants(p<0.001). The majority 316 (54.48%) of participants in the treat-all cohort began treatment at baseline WHO stage II, whereas more than two-thirds (66.55%) of pre-treat all cohort participants had initiated treatment at baseline WHO stage III. Regarding Prophylaxis of opportunistic infection (94.69% vs. 93.62%) participants received CPT respectively. More patients in the treat-all cohort started ART when they were functionally working (54.51% vs. 62.59%, p <0.001) (Table 2).

**Table 2:** Baseline clinical and treatment status of adult HIV patients on ART before and after treat all strategies in Hawassa City between Jan.2012 and Dec.2021.

Variables	Category	Pre-treat-all n = 565	Treat-all n = 580	p-value
Baseline CD4 count	<200 cell/m3	299 (52.92)	71 (12.24)	**<0.001**
	201–350 cell/m3	233 (41.24)	196 (33.79)	
	>350 cell/m3	33 (5.84)	313 (53.97)	
WHO stage	I	13(2.30)	203(35.0)	**<0.001**
	II	117(20.71)	316(54.48)	
	III	376(66.55)	37(6.37)	
	IV	59(10.44)	24(4.14)	
Opportunistic infection	Yes	188(33.27)	191(32.93)	0.902
	No	377(66.73)	389(67.07)	
Baseline TB	Yes	45 (7.96)	46(7.93)	0.728
	No	520 (92.04)	534(92.7)	
Baseline functional status	Working	308(54.51)	364 (62.76)	**<0.001**
	Ambulatory	126 (22.30)	187(32.24)	
	Bedridden	131(23.19)	29(5.00)	
CPT given	Yes	535 (94.69)	543 (93.62)	0.262
	No	30 (5.31)	37 (6.38)	
IPT given	Yes	481 (85.13)	546(94.14)	**<0.001**
	No	84 (14.87)	34(5.86)	
Initial Regimen	TDF-3TC-EFV	372(65.84)	379(65.34)	1.00
	AZT-3TC-NVP	118(20.88)	119(20.51)	
	AZT-3TC-EFV	48(8.50)	52(8.96)	
	TDF-3TC-NVP	27(4.78)	30(5.17)	
Drug adherence	Good	384(67.96)	538(92.76)	**<0.001**
	Fair	62(10.97)	26(4.48)	
	Poor	119(21.06)	16 (2.76)	
Baseline BMI	18.5–24.9	424(75.04)	436 (75.17)	0.770
	<18.5	129(22.83)	130 (22.41)	
	>or = 25	12 (2.12)	14 (2.41)	
Comorbidity	Yes	172(30.44)	176(30.34)	0.971
	No	393(69.56)	404(69.66)	

### The incidence of LTFU

**Fig 1:**
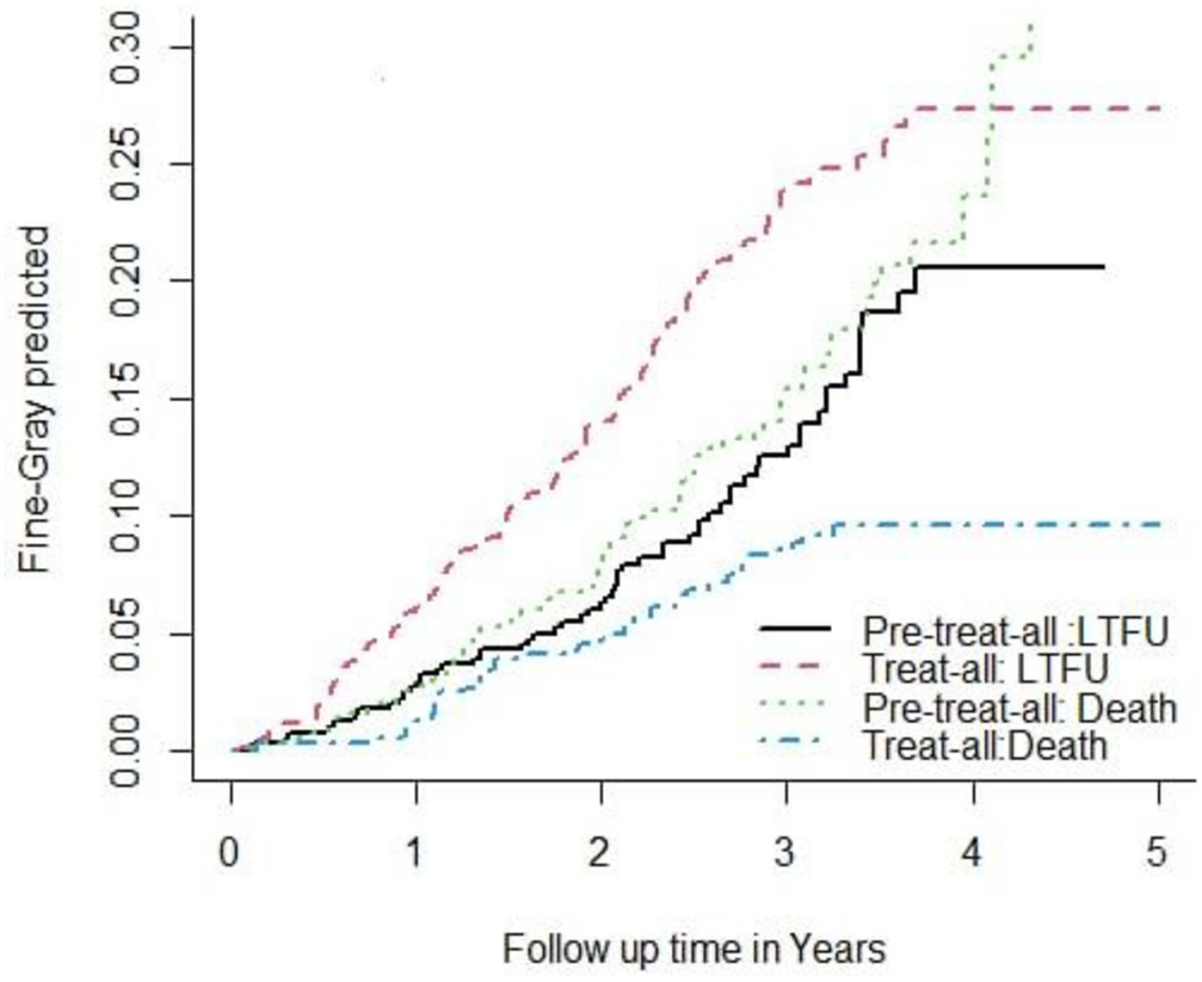
The cumulative incidence curve of LTFU and death of adult HIV patients by treatment strategy (pre-treat-all versus treat-all) in Hawassa City July 2022.

The participants were followed for 1279.27 and 1406.78 person-years. The median follow-up was 26.49 months (IQR: 17.03–37.93) and 29.53 months (IQR: 17.16–40.38) for the pre-treat-all and treat-all cohorts, respectively. In the pre-treat-all cohort, 63 (11.15%; 95% CI: 8.8, 14) lost, 75 (13.27%; 95% CI: 10.7, 16.3) died, and 427 (75.58%; 95% CI: 71.8, 78.9) were censored during the five-year study period. While in the treat-all cohort, 122 (21.03%; 17.9, 24.5) lost, 43 (7.41%; 95% CI: 5.5, 9.8) died, and 415 (71.5; 95% CI: 67.7, 75.0) were censored. The cumulative incidence of LTFU was 4.92(3.84,6.3) and 8.67(7.26,10.3) per 100 person-years (PY) in pre-treat all and treat all cohorts, respectively. The cumulative incidence of mortality was 5.86(4.67,7.35) and 3(2.26,4.12) per 100 PY in pre-treat and treat all cohorts, respectively (Fig 1).

**Fig 2:**
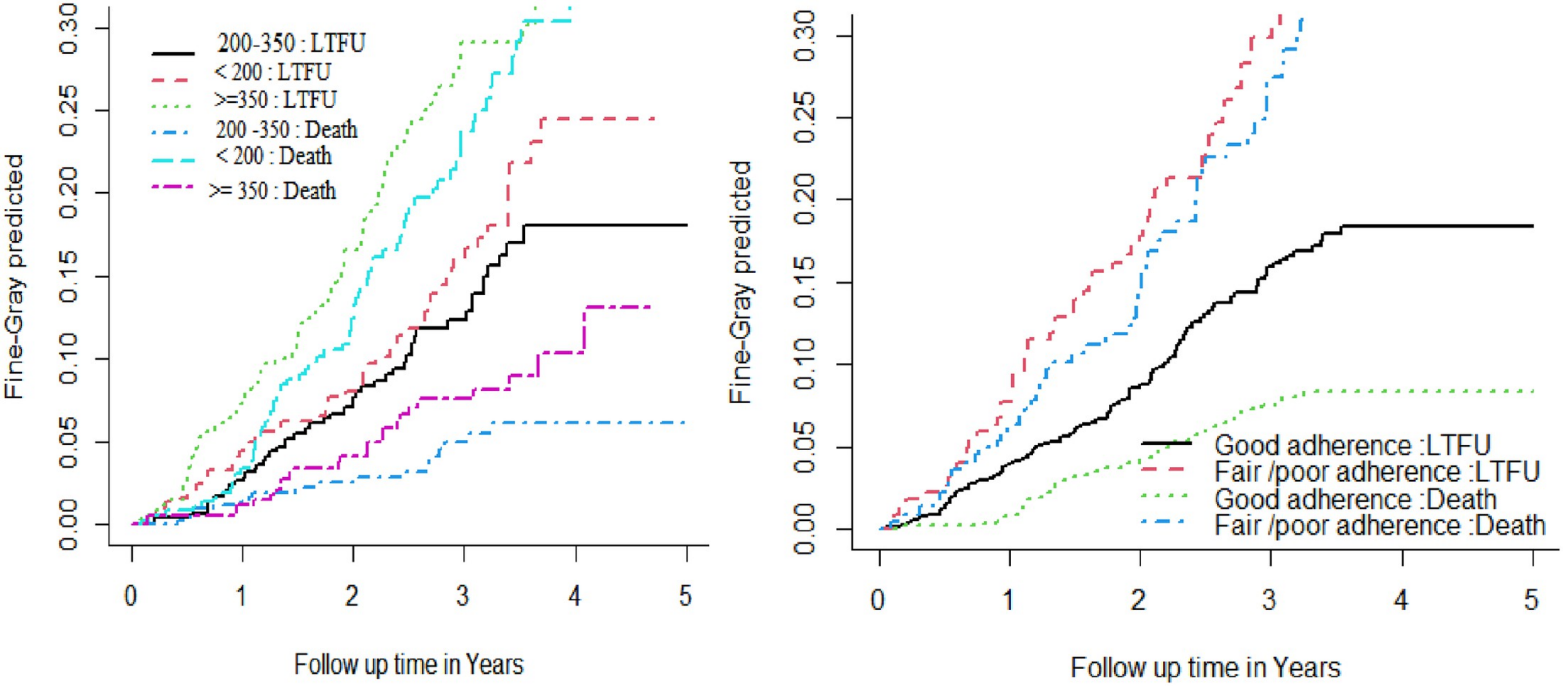
The cumulative incidence function of LTFU and death based on adherence to care and CD4 cell count at ART initiation using ART data of public health facilities in Hawassa City Jan 2012 to Dec 2021.

The incidence of LTFU in patients with baseline CD4 counts > 350 cell/m3 was 10 per 100 person-year observation it was 6.5 and 4.7 per 100 person-year for CD4 < 200 cell/m3 and 200–350 cell/m3, respectively. While the incidence of mortality among patients with baseline CD4 counts < 200 cell/m3 was 9.2 per 100 person-year observation it was 3 and 1.8 per 100 person-days for CD4 >350 cell/m3 and 200–350 cell/m3, respectively (Fig 2).

**Fig 3:**
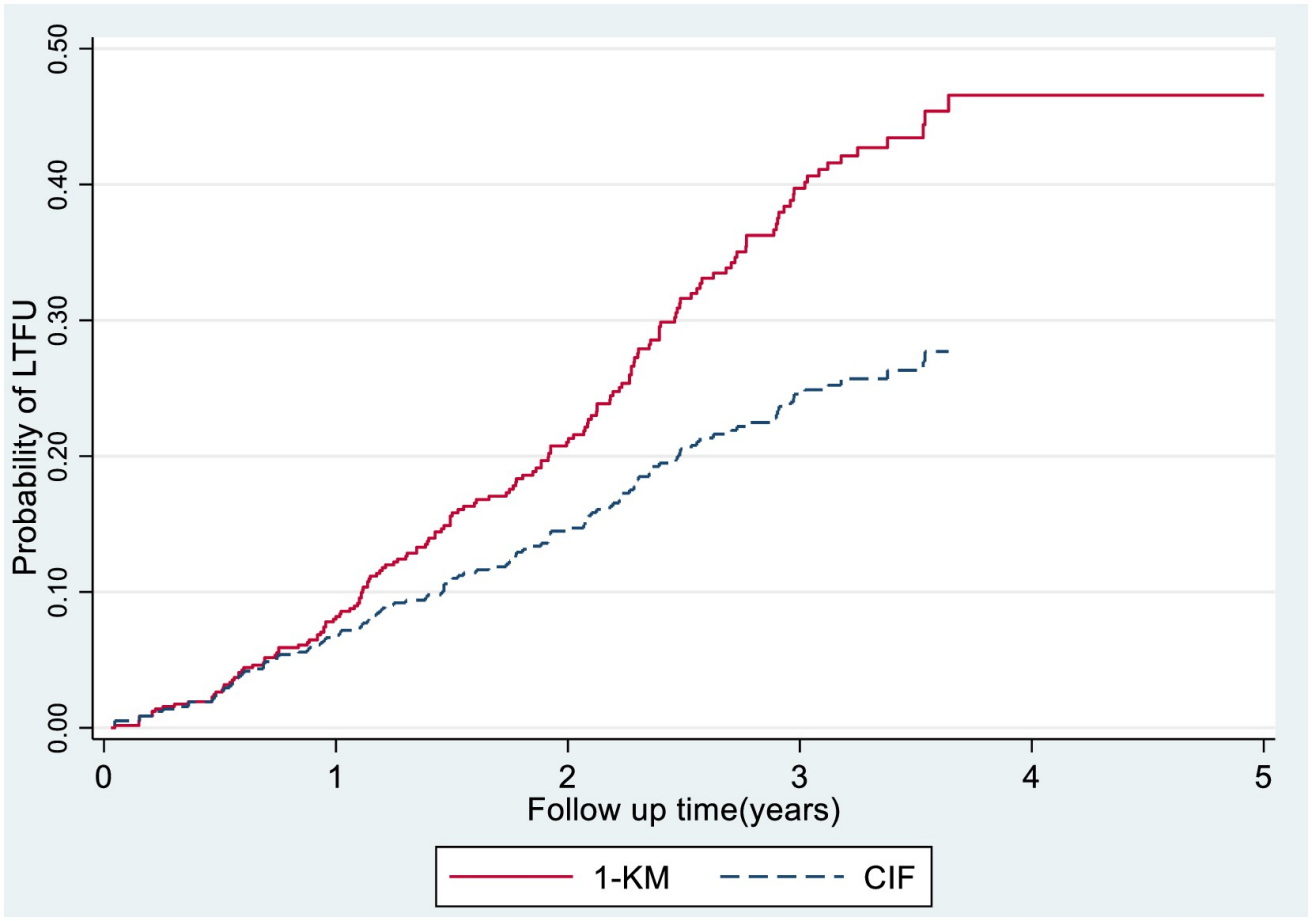
The estimate of cumulative incidence curves of LTFU by standard Cox model and competing risk regression model.

The difference between the two estimates of the cumulative probability of LTFU (one minus Kaplan—Meier and cumulative incidence by considering competing for the effect of death) showed that the 1-km from the standard Cox model overestimates the probability of experiencing LTFU at each subsequent follow-up time, except in the case when there is no competing event at the beginning of the follow-up period (Fig 3).

### Predictors of LTFU in pre-treat-all cohort

In multivariable competing risk analysis revealed: that the sub-hazard of LTFU among adults whose ART drug adherence was fair/poor was about 5 times higher than those with good adherence (aSHR: 5.17; (95% CI 1.97, 13.51). The sub-hazard of LTFU among adults who started ART at baseline WHO stage III/ IV was 2.7 times higher than those who started with baseline WHO stage I/II (aSHR:2.69; 95% CI: 1.27, 5.71). The sub-hazard of LTFU among adults who did not take CPT was 3 times higher than those who took CPT (aSHR: 3.05; (95% CI 1.27, 7.34). Furthermore, the sub-hazard of LTFU was about 2 times higher among adults who started ART with baseline underweight (BMI<18.5) compared to those who started at normal nutritional status (BMI = 18.5–24.999) (aSHR:2.13; 95% CI: 1.15–3.93) (Table 3).

**Table 3:** Bivariable and multivariable competing risk regression for predictors of LTFU among adult HIV patients taking ART in pre-treat all period in Hawassa city public health facilities, Hawassa, Ethiopia, 2022.

Variables	Category	pre-treat all				
		Survival status			cSHR (95% CI)	aSHR (95% CI)
		Death	LTFU	Censored		
Marital status	Single	18	29	89	3.08(1.77,5.33) **	0.95(0.40,2.23)
	Married	27	23	281	1	1
	Divorced	30	11	57	1.36(.67, 2.77)	0.51(0.21,1.24)
Disclosure	Yes	56	27	369	1	1
	No	19	36	58	6.10(3.72,10.0) **	2.82(0.60,13.7)
Base functional status	Working	8	15	285	1	1
	Ambulatory	12	15	99	2.27 (1.10, 4.66) *	1.0(0.44,2.38)
	Bedridden	55	33	43	4.13(2.25,5.57) **	0.88(0.37,2.09)
CPT given	Yes	72	48	415	1	
	No	3	15	12	6.36(3.65,11.0) **	3.05(1.27,7.34)
IPT given	Yes	58	43	380	1	1
	No	17	20	47	2.84(1.68,4.80) **	0.64(.27,1.54)
Drug Adherence	Good	13	12	359	1	
	Fair or poor	62	51	68	8.70(4.64,16.29) **	5.17(1.97,13.5) **
Baseline WHO stage	stage I or II	15	8	165	1	1
	stage III or IV	60	55	262	2.96(1.40, 6.23) *	2.69(1.27,5.71) *
Baseline CD4 count cell/m3	<200	59	42	198	1.77(1.03,.3.04)	1.34(.67,2.67)
	201–350	10	19	204	1	1
	>350	6	2	25	0.78(.17,3.45)	0.54(0.12,2.34)
Age	15–24	18	53	152	0.52 (.28,.94) *	1.45(0.61,3.43)
	25–34	31	68	280	0.31 (.16,.61) *	2.00(0.61,6.55)
	35–44	34	41	265	0.27 (.10,.68) *	1.46(0.38,5.63)
	>/ = 45	35	20	148	1	1
BMI	Normal	34	25	361	1	1
	Underweight	40	37	57	4.33(2.61, 7.20) *	2.13(1.15,3.93) **
	Overweight	1	1	9	1.49(.20–10.85)	1.87 (0.32,10.8)
Caregiver	Yes	55	26	365	1	1
	No	20	37	62	5.77(3.51,9.49) **	1.15(0.24,5.35)
Cell phone	Yes	59	38	347	1	
	No	16	25	80	2.55(1.54,4.21) **	0.81(0.38,1.72)

**significant at p-value <0.001

*significant at p-value <0.05,

CSHR = crude sub hazard ratio, ASHR = adjusted sub hazard ratio.

### Predictors Of LTFU in treat-all cohort

In multivariable competing risk analysis revealed: that the sub-hazard of LTFU among patients whose ART drug adherence was fair/poor was 2 times higher than those with good adherence (aSHR: 2.07; 95% CI: 1.18, 3.65). The sub-hazard of LTFU among adults who started ART at baseline underweight (BMI<18.5) was 1.71 times higher compared to those who started at normal nutritional status (BMI = 18.5–24.999) (aSHR:1.71; 95% CI: 1.13, 2.56). Furthermore, the sub-hazard of LTFU among adults who started ART at baseline CD4 cell counts of >350cell/m3 was 1.61 times higher than those started at baseline CD4 cell count of 201-350cell/m3 (aSHR: 1.67; 95% CI: 1.05–2.65) (Table 4).

**Table 4:** Bivariable and multivariable competing risk regression analysis for predictors of LTFU among adult HIV patients on ART in treat-all periods in Hawassa city public health facilities from Jan1, 2017 to Dec 31, 2021.

Variables	Category	Treat all				
		Survival status			cSHR (95% CI)	aSHR (95% CI)
		Death	LTFU	Censored		
Sex	Male	19	62	185	1.26 (0.8, 1.79) *	1.28 (.88, 1.85)
	Female	24	60	230	1	1
Marital status	Single	22	42	146	0.83(0.56, 1.23)	0.83(.56, 1.23)
	Married	17	66	207	1	1
	Divorced	4	14	62	0.68(.38, 1.19) *	0.72(.39, 1.33)
Disclosure	Yes	36	106	388	1	1
	No	7	16	27	1.49 (0.90,2.46) *	1.20(.68, 2.12)
Base functional status	Working	29	69	266	1	1
	Ambulatory	10	42	135	1.25 (.85, 1.85)	1.27 (.83, 1.92)
	Bedridden	4	11	14	2.22(1.20,4.11) **	1.97 (.80, 4.88)
CPT given	Yes	4	13	23	1	
	No	39	109	392	1.91 (1.09,3.34) *	1.21(.58, 2.50)
IPT given	Yes	5	10	18	1	
	No	38	112	397	1.60 (.85, 3.01) *	1.12 (.45, 2.77)
Drug adherence	Good	42	106	390	1	
	Fair or poor	1	16	25	2.10 (1.25,3.54) *	2.07(1.18, 3.65) *
Baseline WHO stage	I or II	31	102	386	1	1
	III or IV	12	20	29	1.67 (1.04, 2.68) *	1.76 (0.96, 3.29)
Baseline CD4 count	<200 cell/m3	16	11	44	0.93(.46,1.84)	0.57(.24, 1.34)
	201-350cell/m3	8	31	157	1	1
	>350 cell/m3	19	80	214	1.71(1.13,2.59) *	1.67 (1.05, 2.65) *
Age	15–24	7	33	97	1.90(1.07, 3.36) *	1.78 (.97, 3.26)
	25–34	7	46	130	1.78 (0.97, 3.27) *	1.75 (.93, 3.28)
	35–44	15	29	102	1.39 (.75, 2.57)	1.35 (.71, 2.57)
	>/ = 45	14	14	86	1	1
BMI	Normal	29	82	325	1	1
	Underweight	13	38	79	1.66(1.13, 2.43) *	1.71(1.13, 2.56) *
	Overweight	1	2	11	0.80(.18, 3.55)	0.89(0.23, 3.68)

**significant at p-value <0.001

*significant at p-value <0.05,

CSHR = crude sub hazard ratio, aSHR = adjusted sub hazard ratio.

## Discussion

This study investigated the incidence and predictors of LTFU in HIV-positive adults initiated on ART before and after the implementation of a test-and-treat strategy. As illustrated in Fig 3, the cumulative incidence function estimates the valid rate of LTFU, adjusting for mortality’s competing effect, as those who die are no longer at risk, which is in line with other similar studies [42, 43]. The estimated rate of LTFU in this study was 4.92 and 8.67 per 100 person-years in the pre-treat-all and treat-all cohorts, respectively.

The incidence of LTFU was 8.67 per 100 person-years in the treat-all period was consistent with 8.9 per 100 person-year reported from North Shewa zone, public hospitals [44]. However, it was lower than the previous studies conducted at Felege Hiwot Comprehensive Specialized Hospital, Northwest Ethiopia [45] and Burundi [46]. The differences might be due to the differences in baseline clinical, the occurrence of opportunistic infection, and nutritional characteristics of the patients, as low immunological status was a key criterion for treatment initiation in the pre-treat-all era, impacting care retention.

The LTFU rate increased significantly in the treat-all strategy compared to the pre-treat-all era, emphasizing the need for ongoing monitoring and follow-up. It is essential to implement a patient-centered approach and transportation assistance to retain patients on the program, particularly those who are more at risk for LTFU [47, 48]. Retention in care is essential for achieving optimal health outcomes and reducing the spread of HIV within communities [49].

This study found the advanced WHO clinical stage of diseases at baseline was the strongest predictor of LTFU. This finding was similar to findings reported in the Hadiya zone, Pawi General Hospital, Arbaminch General Hospital, and Gondar University Hospital [4, 15, 30]. This can be explained by the fact that in advanced WHO clinical disease, patients with lowered immunity face increased opportunistic infections and are bedridden, leading to severe illness and unreported deaths.

Underweight individuals are at a higher risk of developing LTFU. This is in line with different previous studies in Ethiopia [20, 50] and Central Kenya [50]. This is because undernourished individuals with HIV and AIDS can progress diseases to severe stages, which increases the occurrence of opportunistic infections. This dual burden can lead to being bedridden and ultimately dying [51].

The sub-hazard of LTFU among respondents with fair or poor adherence was higher than among respondents with good adherence. This is comparable to findings from other similar studies [15, 19]. The reason for this may be due to low awareness of lifelong drug adherence and insufficient physiological preparation for long-term treatment follow-up, which may result in high viral loads, treatment failure, increased risk of OIs, and ultimately mortality in ART patients [6]. Therefore, maintaining adherence and retention in care is crucial to determining the optimal benefits of ART programs.

Furthermore, individuals who started ART with a high CD4 count were more likely to develop LTFU. The finding was in line with other similar studies [52, 53]. However, this finding is in contrast to many studies that reported poor immunologic status (low CD4+ cell count) at ART initiation as a predictor of increased hazard of LTFU [12, 14, 54, 55]. Patients with a good immunological state may fail to adhere to early induction programs because they feel well and do not think about their medication needs. Lack of psychological preparation before treatment initiation could also be a factor. Therefore, targeted interventions and support may be needed to reduce LTFU and promote the potential benefits of early ART introduction at higher CD4 counts.

### Limitations of the study

The findings of this study might suffer from the fact that it is a retrospective study. Predictors like viral load, hemoglobin level, clinical factors, institution-level factors, and contextual factors were not included as they were not well documented on patient cards. Moreover, the effect of silent transfer out and unreported death was not considered, and the distance from health facilities was reported as one factor for loss to follow-up but not addressed.

### Conclusion

The findings of this study showed that the incidence of loss to follow-up was significantly higher during the treat-all era than in the pre-treat-all era. Underweight (BMI <18.5), WHO stages III and IV, fair or poor adherence, and CD4 cells > 350 cells in the treat-all cohort were independent predictors of loss to follow-up, as were underweight (BMI <18.5), fair or poor adherence, and underweight in the pre-treat all cohort. Therefore, targeted interventions, such as nutritional support and strengthening medication adherence counseling, should be implemented to maintain treatment retention and reduce antiretroviral therapy dropout rates.

## Supporting information

S1 Data (DTA)

S1 Checklist (DOCX)

S1 Questionnaire (DOCX)

S1 Text (DOCX)

## Abbreviations

AIDS: Acquired Immunodeficiency Syndrome
ART: Antiretroviral therapy
CD4: Cluster of Differentiation 4
CPT: Cotrimoxazole prophylactic therapy
IPT: Isoniazid preventive therapy
LTUF: loss to follow up
sAHR: sub-distributed Adjusted hazard ratio
