# Supplementary material for: Predictors of loss to follow up among adults on antiretroviral therapy before and after the start of treat-all strategy in public health facilities of Hawassa city, Ethiopia: A Competing risk regression
Source: PLoS One. 2024 Mar 14;19(3):e0299505. doi: 10.1371/journal.pone.0299505 (PMC10939213; doi:10.1371/journal.pone.0299505)
Supplement: S1 Checklist — (DOCX) [file pone.0299505.s002.docx]

STROBE Statement—checklist of items that should be included in reports of observational studies

|  | Item No. | Recommendation | Page  No. | Relevant text from manuscript |
| --- | --- | --- | --- | --- |
| **Title and abstract** | 1 | (*a*) Indicate the study’s design with a commonly used term in the title or the abstract | 1 | Retrospective Follow-up Study. |
|  |  | (*b*) Provide in the abstract an informative and balanced summary of what was done and what was found | 1 | An institution-based retrospective follow-up study was conducted among 1190 randomly selected adults on antiretroviral therapy in public health facilities in Hawassa City  A Gray’s test and cumulative incidence curve were used to compare the cumulative incidence function of loss to follow-up.  Result: The cumulative incidence of lost-to-follow-up was 4.92(3.84,6.3) and 8.67(7.26,10.3) per 100 person-years (PY) in pre-treat all and treat all cohorts, respectively.  Fair/poor adherence (aSHR:5.17; (95% CI 1.97, 13.51), underweight (aSHR:2.13; 95% CI: 1.15-3.93) and WHO stage III/IV (aSHR:2.69; 95% CI: 1.27, 5.71) were predictors of loss up in pre–treat all, whereas fair/poor adherence (aSHR = 2.07; 95% CI: 1.18, 3.68), underweight (aSHR:1.71; 95% CI: 1.13, 2.56), and CD4 cell >350 cell/m3 (aSHR: 1.67; 95% CI: 1.05, 2.65) predicts of loss up in treat all cohorts. |
| Introduction | | | |  |
| Background/rationale | 2 | Explain the scientific background and rationale for the investigation being reported | 3, 4 |  |
| Objectives | 3 | State specific objectives, including any prespecified hypotheses | 4 | To assess the incidence of loss to follow-up before and after the start of the treat-all strategy among adults on anti-retroviral therapy in public health facilities in Hawassa City, Ethiopia  To identify the predictors of LTFU before and after the start of the treat-all strategy among adults on anti-retroviral therapy in public health facilities in Hawassa City, Ethiopia |
| Methods | | | |  |
| Study design | 4 | Present key elements of study design early in the paper | 4 |  |
| Setting | 5 | Describe the setting, locations, and relevant dates, including periods of recruitment, exposure, follow-up, and data collection | 4, 5, and 6 |  |
| Participants | 6 | (*a*) *Cohort study*—Give the eligibility criteria, and the sources and methods of selection of participants. Describe methods of follow-up  *Case-control study*—Give the eligibility criteria, and the sources and methods of case ascertainment and control selection. Give the rationale for the choice of cases and controls  *Cross-sectional study*—Give the eligibility criteria, and the sources and methods of selection of participants | 5, 6 |  |
|  |  | (*b*) *Cohort study*—For matched studies, give matching criteria and number of exposed and unexposed  *Case-control study*—For matched studies, give matching criteria and the number of controls per case |  |  |
| Variables | 7 | Clearly define all outcomes, exposures, predictors, potential confounders, and effect modifiers. Give diagnostic criteria, if applicable | 6 | **Event:** loss to follow-up (LTFU) defined as not taking an ART refill for three months or longer from the last attendance for the refill and not yet classified as dead or transferred out |
| Data sources/ measurement | 8* | For each variable of interest, give sources of data and details of methods of assessment (measurement). Describe comparability of assessment methods if there is more than one group | 6,7 |  |
| Bias | 9 | Describe any efforts to address potential sources of bias |  |  |
| Study size | 10 | Explain how the study size was arrived at | 5 |  |

Continued on next page

| Quantitative variables | 11 | Explain how quantitative variables were handled in the analyses. If applicable, describe which groupings were chosen and why |  |  |
| --- | --- | --- | --- | --- |
| Statistical methods | 12 | (*a*) Describe all statistical methods, including those used to control for confounding | 7 |  |
|  |  | (*b*) Describe any methods used to examine subgroups and interactions |  |  |
|  |  | (*c*) Explain how missing data were addressed |  |  |
|  |  | (*d*) *Cohort study*—If applicable, explain how loss to follow-up was addressed  *Case-control study*—If applicable, explain how matching of cases and controls was addressed  *Cross-sectional study*—If applicable, describe analytical methods taking account of sampling strategy |  |  |
|  |  | (*e*) Describe any sensitivity analyses |  |  |
| Results | | | | |
| Participants | 13* | (a) Report numbers of individuals at each stage of study—eg numbers potentially eligible, examined for eligibility, confirmed eligible, included in the study, completing follow-up, and analysed |  |  |
|  |  | (b) Give reasons for non-participation at each stage |  |  |
|  |  | (c) Consider use of a flow diagram |  |  |
| Descriptive data | 14* | (a) Give characteristics of study participants (eg demographic, clinical, social) and information on exposures and potential confounders | 8 |  |
|  |  | (b) Indicate number of participants with missing data for each variable of interest |  |  |
|  |  | (c) *Cohort study*—Summarise follow-up time (eg, average and total amount) | 11 |  |
| Outcome data | 15* | *Cohort study*—Report numbers of outcome events or summary measures over time | 11 | In the pre-treat-all cohort, 63 (11.15%; 95% CI: 8.8, 14) lost, 75 (13.27%; 95% CI: 10.7, 16.3) died, and 427 (75.58%; 95% CI: 71.8, 78.9) were censored during the five-year study period. While in the treat-all cohort, 122 (21.03%; 17.9, 24.5) lost, 43 (7.41%; 95% CI: 5.5, 9.8) died, and 415 (71.5; 95% CI: 67.7, 75.0) were censored |
|  |  | *Case-control study—*Report numbers in each exposure category, or summary measures of exposure |  |  |
|  |  | *Cross-sectional study—*Report numbers of outcome events or summary measures |  |  |
| Main results | 16 | (*a*) Give unadjusted estimates and, if applicable, confounder-adjusted estimates and their precision (eg, 95% confidence interval). Make clear which confounders were adjusted for and why they were included |  |  |
|  |  | (*b*) Report category boundaries when continuous variables were categorized |  |  |
|  |  | (*c*) If relevant, consider translating estimates of relative risk into absolute risk for a meaningful time period |  |  |

Continued on next page

| Other analyses | 17 | Report other analyses done—eg analyses of subgroups and interactions, and sensitivity analyses |  |  |
| --- | --- | --- | --- | --- |
| Discussion | | | | |
| Key results | 18 | Summarise key results with reference to study objectives | 15 |  |
| Limitations | 19 | Discuss limitations of the study, taking into account sources of potential bias or imprecision. Discuss both direction and magnitude of any potential bias | 17 |  |
| Interpretation | 20 | Give a cautious overall interpretation of results considering objectives, limitations, multiplicity of analyses, results from similar studies, and other relevant evidence | 15-17 |  |
| Generalisability | 21 | Discuss the generalisability (external validity) of the study results | 15-18 |  |
| Other information | |  | | |
| Funding | 22 | Give the source of funding and the role of the funders for the present study and, if applicable, for the original study on which the present article is based | 24 | Funding not applicable. |

*Give information separately for cases and controls in case-control studies and, if applicable, for exposed and unexposed groups in cohort and cross-sectional studies.

**Note:** An Explanation and Elaboration article discusses each checklist item and gives methodological background and published examples of transparent reporting. The STROBE checklist is best used in conjunction with this article (freely available on the Web sites of PLoS Medicine at http://www.plosmedicine.org/, Annals of Internal Medicine at http://www.annals.org/, and Epidemiology at http://www.epidem.com/). Information on the STROBE Initiative is available at www.strobe-statement.org.
