# Supplementary material for: Predictors of loss to follow up among adults on antiretroviral therapy before and after the start of treat-all strategy in public health facilities of Hawassa city, Ethiopia: A Competing risk regression
Source: PLoS One. 2024 Mar 14;19(3):e0299505. doi: 10.1371/journal.pone.0299505 (PMC10939213; doi:10.1371/journal.pone.0299505)
Supplement: S1 Questionnaire — (DOCX) [file pone.0299505.s003.docx]

Data collection checklist

| No | | | Sociodemographic variables | Possible answers | | | | | | | | | Skip |
| --- | --- | --- | --- | --- | --- | --- | --- | --- | --- | --- | --- | --- | --- |
| 101 | | | Age at enrollment | ……………. years | | | | | | | | |  |
| 102 | | | Date of enrollment | ……/……/ …….dd/mm//yy | | | | | | | | |  |
| 103 | | | Sex | 1. Male  2. female | | | | | | | | |  |
| 104 | | | Religion | 1. Orthodox  3. Catholic  2. Muslim  4. Protestant  5. Other specify---- | | | | | | | | |  |
| 105 | | | Residence | 1.Urban  2.Rural | | | | | | | | |  |
| 106 | | | Marital status | 1. Single  2. Married  3. Divorced  4. Widowed  5. Separated | | | | | | | | |  |
| 107 | | | Education | 1. can’t able to read & write 2. Read and write   3.Elementary  4.Secondary  5. Collage & University | | | | | | | | |  |
| 108 | | | Occupation | 1. Daily laborer 2. Farmer 3. merchant 4. driver 5. Student 6. house wife 7. Government   employee   1. Others,   specify ------- | | | | | | | | | |
| 109 | | | Have you a caregiver? | 1.yes  2. No | | | | | | | | |  |
| 110 | | | Disclosure status | 1.Disclosed  2.Not disclosed  3.Unknown | | | | if no go to 112 | | | | | |
| 111 | | | To whom disclosed | 1. Spouse (wife/husband) 2. Parents 3. Relatives 4. Siblings 5. children’s 6. Others-- | | | | | | | | |  |
| 112 | | | Spouse HIV status | 1. Positive 2. Negative 3. Not recorded 4. Unknown 5. Not applicable | | | | | | | | |  |
| 113 | | | Having cell phone | 1. Yes 2. No | | | | | | | | |  |
| Part2 | Base line clinical, laboratory and ART information | | | | | Possible answers | | | | | Skip | | |
| 114 | opportunistic infections at enrollment | | | | | 1.Yes  If yes, Specify___  2. No | | | | |  | | |
| 115 | Weight at base line | | | | | ……………kg | | | | |  | | |
| 116 | Height/length at base line | | | | | ……….cm | | | | |  | | |
| 117 | Functional status at baseline | | | | | 1.Working  2.Ambulatory  3.Bedridden | | | | |  | | |
| 118 | WHO clinical stage at base line | | | | | 1.Stage I  2.Stage II  3.StageIII  4.Stage IV | | | | |  | | |
| 119 | Baseline CD4 count | | | | | ……….cells/ml | | | | |  | | |
| 120 | First Viral load if done | | | | | ……..copies/ml | | | | |  | | |
| 121 | Baseline Hgb | | | | | …….g/dl | | | | |  | | |
| 122 | Previous TB treatment | | | | | 1. 1. Yes , If yes date ------- Treatment outcome-------- 2. 2. No 3. 3. Not recorded | | | | |  | | |
| 123 | Current TB status | | | | | 1. Positive 2. Negative 3. Unknown 4. Not recorded | | | | |  | | |
| 124 | Previous disease Comorbidities (HTN,DM, Stroke, CA, others) | | | | 1. Yes, if yes specify........... 2. No | | | | | |  | | |
| Part3 | ART treatment and other Medications information | | | | Possible answers | | | | | | Skip | | |
| 125 | ART start date | | | | ……../……../……..dd/mm/yy | | | | | |  | | |
| 126 | OI prophylaxis given | | | | 1.Not given  2.Co-trimoxazole  3.INH  4.Others specify- | | | | | |  | | |
| 127 | Type of regimen when  start (original regimen) | | | | 1. 4a=d4t-3TC-NVP  2. 4b=d4t-3TC-EFV  3.4c=AZT-3TC-NVP  4.4d=AZT-3TC-EFV  5.4e=TDF-3TC-EFV  6.4h=ABC-3TC-NVP  7.4g=ABC-3TC-EFV  8. 2nd line regimens  9. Others specify----- | | | | | |  | | |
| Part4 | Patient follow up information (filled from ART follow up form) recent results | | | | Possible answers | | | | | | Skip | | |
| 128 | Date confirmed HIV positive | | | | ………/………../……..dd/mm/yy | | | | | |  | | |
| 129 | Co-trimoxazole preventive therapy | | | | 1. Given   2. Not given | | | | | | |  | |
| 130 | Recent ARV adherence | | | | 1. Good  2. Fair  3. Poor | | | | | If good skip to Q 137 | | | |
| 131 | Reason for fair/poor adherence | | | | 1. Toxicity/SE  2. Share with others  3. Forgot  4. Felt better  5. Too ill  6. Stigma  7. Drug stoke out  8. Travelling problem  9. Depression  10. Others specify---- | | | | | | | | |
| 133 | | Does the regimen change | | | | 1. Yes 2. No | If no, skip to Q140 | | | | | | |
| 134 | | If yes, reason for regimen change | | | | 1. Toxicity/SE  2.New drug available  3. Drug out of stoke  4. Clinical failure  5.Immunologic failure  6. Virologic failure  7. New TB  8.Other specify | | |  | | | | |
| 135 | | Current status | | | | 1. On follow up  2. Dead  3.Lost follow up  4. transfer to other health facility | | |  | | | | |
| 136 | | If lost to follow up when? | | | | ………/………../……..dd/mm/yy | | |  | | | | |
| 137 | | If dead or transfer to other facility when? | | | | ………/………../……..dd/mm/yy | | |  | | | | |
| 138 | | Date at last follow up | | | | ………/………../……..dd/mm/yy | | |  | | | | |
