# Supplementary material for: Predictors of loss to follow up among adults on antiretroviral therapy before and after the start of treat-all strategy in public health facilities of Hawassa city, Ethiopia: A Competing risk regression
Source: PLoS One. 2024 Mar 14;19(3):e0299505. doi: 10.1371/journal.pone.0299505 (PMC10939213; doi:10.1371/journal.pone.0299505)
Supplement: S1 Text — (DOCX) [file pone.0299505.s004.docx]

**Test of proportional-hazards assumption of pre-treat all cohort (****Global test)**

**Test of proportional-hazards assumption all cohort (Global test)**
